# Synapse elimination in the developing cerebellum

**DOI:** 10.1007/s00018-013-1405-2

**Published:** 2013-06-28

**Authors:** Kouichi Hashimoto, Masanobu Kano

**Affiliations:** 1 Department of Neurophysiology, Graduate School of Biomedical Sciences, Hiroshima University, 1-2-3 Kasumi, Minami-ku, Hiroshima, 734-8551 Japan; 2 PRESTO, Japan Science and Technology Agency, Saitama, 332-0012 Japan; 3 Department of Neurophysiology, Graduate School of Medicine, The University of Tokyo, 7-3-1 Hongo, Bunkyo-ku, Tokyo, 113-0033 Japan

**Keywords:** Dendrite, Synapse, Cerebellum, Climbing fiber, Parallel fiber, Purkinje cell, Inferior olive, Postnatal development

## Abstract

Neural circuits in neonatal animals contain numerous redundant synapses that are functionally immature. During the postnatal period, unnecessary synapses are eliminated while functionally important synapses become stronger and mature. The climbing fiber (CF) to the Purkinje cell (PC) synapse is a representative model for the analysis of postnatal refinement of neuronal circuits in the central nervous system. PCs are initially innervated by multiple CFs with similar strengths around postnatal day 3 (P3). Only a single CF is selectively strengthened during P3–P7 (functional differentiation), and the strengthened CF undergoes translocation from soma to dendrites of PCs from P9 on (dendritic translocation). Following the functional differentiation, supernumerary CF synapses on the soma are eliminated, which proceeds in two distinct phases: the early phase from P7 to around P11 and the late phase from around P12 to P17. Here, we review our current understanding of cellular and molecular mechanisms of CF synapse elimination in the developing cerebellum.

## Introduction

Formation of precise neuronal connections during development is a prerequisite for proper functions of the nervous system. At birth, neuronal connections are redundant, but they are refined and become functionally mature through activity-dependent competition among redundant synaptic inputs to each postsynaptic neuron. During the postnatal period, functionally important synapses are selectively strengthened and stabilized, whereas unnecessary surplus connections are weakened and eventually eliminated. Many studies indicate that these processes are dependent on neural activity particularly during the limited postnatal period known as the ‘critical period’ or ‘sensitive period’ [1–5].

The climbing fiber (CF) to the Purkinje cell (PC) synapse in the cerebellar cortex is regarded as a representative model system for analyzing the mechanisms for developmental establishment of the functional connections in the central nervous system. In the adult cerebellum, each PC is innervated by a single CF (mono innervation) originating from the inferior olive of the contralateral medulla oblongata. Each CF forms hundreds of synaptic contacts on the proximal part of PC dendrites [6, 7]. Therefore, activation of a single CF causes strong depolarization that triggers a Ca^2+^ transient due to activation of voltage-dependent Ca^2+^ channels (VDCCs) in PC dendrites [8]. In early postnatal days, however, all PCs are innervated by multiple CFs with weak synaptic responses (multiple innervation) [9–11]. Surplus CFs are eventually eliminated during postnatal development, and mono innervation is attained by the end of the third postnatal week in mice [12–16]. In this review article, we will integrate our current knowledge and provide an overview of the mechanisms of CF synapse elimination in the developing cerebellum.

## Synaptogenesis of CFs to immature PCs

The cerebellar cortex is known to be subdivided into longitudinally elongated parasagittal bands [17–19]. PCs in each subdivision are innervated by neurons in distinct subnuclei of the inferior olive [20–22]. These parasagittal bands are further subdivided into smaller units called microzones, in each of which PCs display high synchronity of complex spike activity [23, 24] and resultant Ca^2+^ transients [25, 26]. In adult rats, each olivocerebellar axon ramifies several times in the cerebellum and gives rise to 6.6 CFs on average [27]. These CFs terminate in one lobule or multiple continuous or discontinuous lobules, but all of them are aligned within a single rostrocaudally oriented area [28].

Olivo-cerebellar axons reach the primitive cerebellum around E18 [29, 30]. Their projections are largely topographic at birth, and are roughly aligned within rostrocaudally oriented areas [31, 32]. However, the typical “climbing fiber” morphology is not observed at this postnatal age. Immature olivo-cerebellar axons extensively ramify in the white matter and granule cell (GC) layer, and give rise to many thick and thin collaterals around PCs. This stage is called the “creeper stage” [33]. At this stage, PCs still have bipolar shapes (called “simple- and complex-fusiform cells” [34]), have just completed their migration, and are organized in a multilayer. Initially, each olivo-cerebellar axon forms about 100 “creeper” fibers [32]. Morphological analyses have demonstrated that the synapse formation of olivary axons on PCs starts from their arrival to the cerebellar cortex [29, 33, 35, 36]. Electrophysiological analyzes indicate that functional olivo-cerebellar synapses are formed on immature PCs around P3. In juvenile rats and mice in vivo, stimulation in the inferior olive after P3 elicits excitatory responses in PCs [37, 38]. However, the responses of juvenile PCs are graded in parallel with the increase in the stimulus strength [10], which indicates that PCs are innervated by multiple olivo-cerebellar axons.

While molecules related to cell identity are differentially expressed in parasagittal bands in developing cerebellum and inferior olivery neurons [39], it remains unclear how such parasagittal organization is formed during development. Several molecules have been proposed but none of them has been proven to provide spatial cues that may direct CF targeting to appropriate cerebellar zones. Future studies should elucidate a molecular logic for constructing the topographic olivo-cerebellar projection during cerebellar development.

## Postnatal refinement of CF to PC synapses

**Fig. 1:**
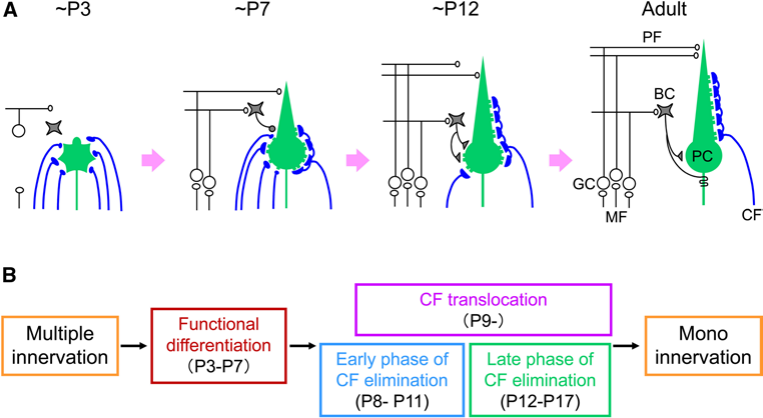
Postnatal refinement of CF to PC synapses. **a** Postnatal development of CF-PC synapses in the mouse. **b** Four distinct phases of postnatal refinement of CF-PC synapses. Modified from Watanabe and Kano [16]

While the microzonal projection is largely established at early postnatal days, each PC is innervated by multiple CFs. PCs are devoid of large primary dendrites and CFs mainly form terminals on the fine processes emerging from the PC somata. Adult-like, mono innervation is gradually established during postnatal development. Studies on the mechanisms underlying postnatal development of the CF to PC synapse were initiated from the analyses of spontaneously occurring mutant animals. Several mutant mice that have malformation of cerebellar cortex (reeler [40]), degeneration of cerebellar GCs (weaver [41, 42]), or impairment of PC morphogenesis and parallel fiber (PF)-PC synaptogenesis (staggerer [43, 44]) show persistent multiple CF innervation of PCs in adulthood. Defects in CF synapse elimination are also observed in mice or rats whose GCs are artificially destroyed by methylazoxy methanol acetate [13], virus infection [45], or X-ray irradiation [46, 47]. These pioneering analyses suggest that proper formation of GCs and PFs is crucial for CF synapse elimination. In particular, one study by Crepel et al. [46] has provided an important concept for the postnatal refinement of neuronal circuits. They demonstrated that, in the hypogranular cerebellum by X-ray irradiation to neonatal rats, CF elimination proceeded properly at first, but further regression after P10 was severely impaired [46]. This result suggests that CF synapse elimination is not a unitary event, but mediated by at least two distinct processes, i.e., the early and late phases that are independent of and dependent on GC generation, respectively [9–11]. Recent analyses have disclosed that the postnatal refinement of CFs proceeds in at least four developmental phases (Fig. 1). Immature PCs are innervated by multiple CFs with similar synaptic strengths at birth. Then, a single CF is selectively strengthened in each PC until P7 (functional differentiation). From P9, only the strengthened CF translocates and expands its innervations to PC dendrites (CF translocation). In parallel, supernumerary CF synapses on PC somata are eliminated through two distinct processes: the ‘early-phase’ from P7 to around P11 and the ‘late phase’ from around P12 to P17.

## Functional differentiation of multiple CFs; selection of a single ‘winner’ CF

Around P2–P3, most PCs are multiply-innervated by four or more CFs. At this postnatal age, individual multiply-innervating CFs elicit excitatory postsynaptic currents (EPSCs) with similar amplitudes. Then, a single CF in each PC undergoes selective strengthening such that it elicits larger EPSCs than do other CFs [48–51]. Quantitative assessments of disparity among amplitudes of multiple CF-EPSCs in individual PCs indicate that this selective strengthening of a single CF occurs from P3 to P7 [49]. This is thought to be the competitive process for the selection of the single CF that will innervate the PC throughout life.

These electrophysiological data in mice are supported by the morphological analyses in rats [32, 52]. Immature axons from the inferior olive initially give rise to terminal fibers creeping around the PCL. These ‘creeping’ type terminal fibers form a relatively small number of en passant swellings. Then, each fiber comes to form aggregated synaptic terminals surrounding the PC somata (‘nest’ type) [32]. This morphological change occurs from P4 to P7 during the same postnatal period as the functional differentiation of multiple CF inputs, suggesting that this transition from creeper to nest type fiber is the morphological basis for the functional differentiation of CFs. At P9, 57 % of VGluT2-positive CF terminals on a PC soma are formed by a single predominant CF [52]. Because each PC is innervated by one predominant CF and on average 2.5 weaker CFs at P9, each weak CF supplies about 17 % of somatic CF terminals. Therefore, the number of terminals formed by a lesser CF should be about 1/3 of that of the predominant CF. This value is roughly identical to the amplitude ratio calculated from EPSC [49], suggesting that the functional differentiation is mainly attained by a selective increase in synapse formation from a single CF among multiple CFs in each PC.

In addition to the increase in the synaptic terminal number, each CF terminal is also functionally strengthened. The EPSCs arising from weaker CFs have been shown to be suppressed by a low-affinity AMPA receptor antagonist, PDA, more potentially than those arising from the strengthened CF [49]. This result suggests that the glutamate transient in the synaptic cleft is smaller at the weaker CF terminals than at the stronger CF terminals. Further electrophysiological analysis suggests that the average number of synaptic vesicles that are fused to the plasma membrane of presynaptic terminal by each action potential is different between the strengthened and weaker CFs. At single synaptic sites of adult mono-innevating CFs, glutamate is released from multiple synaptic vesicles (multivesicular release) [53]. In multiply innervated PCs, the strongest CF shows clear multivesicular release, but the degree of multivesicular release is smaller at terminals of weaker CFs [49]. Because multivesicular release and resulting large glutamate transient make synaptic transmission reliable, the difference in the degree of multivesicular release can contribute to stabilization of the strengthened CF inputs.

Hashimoto et al. [54] have recently demonstrated that Cav2.1, the pore-forming component of the P/Q type VDCC, is crucial for the biased strengthening of a single CF. As mentioned above, a single CF gains biased strengthening in each PC during the first postnatal week. In the mutant mice in which Cav2.1 in postsynaptic PCs was selectively deleted, the strengthening was not biased to a single CF but multiple CFs were non-selectively strengthened until around P7 [54]. This result suggests that the strengthening of the CF synaptic efficacy and biasing the competition towards a single input are mediated by different mechanisms. Molecular bases are currently unknown, but it is assumed that multiple CFs compete for a limited resource that is provided by PCs and necessary for growth and maintenance of CF synapses. As the amount of the resource increases with postnatal development, the total synaptic efficacy of multiple CFs becomes larger. This process itself is independent of P/Q-type VDCC in PCs, but the assignment of the resource to a single CF may be dependent on P/Q-type VDCC-mediated activity. Stronger CFs can activate postsynaptic P/Q-type VDCC more effectively [48, 49, 55], and may gain more resource than weaker CFs. Importantly, Bosman et al. [48] and Ohtsuki et al. [50] reported that Ca^2+^-dependent long-term potentiation could be induced only in the CF inputs that elicited large excitatory postsynaptic potentials in early postnatal stage. Such activity-dependent competition may eventually result in selective strengthening of a single “winner” CF and weakening of the rest of CFs in each PC.

## CF translocation; expansion of CF innervation to PC dendrites

The territory of CF synapses on PCs expands from soma to dendrites during postnatal development [7, 56, 57], which is known as “CF translocation”. As mentioned above, immature axons from inferior olivary neurons reach the cerebellar cortex around E18 (creeper stage). Around P5, PCs are in the stage of “stellate cells” with the extensive protrusions [34, 39]. From P5 to P9, CFs establish synaptic contacts with the abundant perisomatic protrusions and thorns, and form a plexus on the lower part of the PC somata (“pericellular nest” stage) [57]. From around P6, the stem dendrite starts to grow into the molecular layer, concurrently with withdrawal of the perisomatic processes. Then, secondary and tertiary dendritic arbors of PCs develop, but the translocation of CFs to dendrites is not commenced at this stage. CFs continue to associate around PC somata and do not elongate processes into the molecular layer until around P9 [31, 52, 58].

The CFs start to extend from soma to main dendrites from P9 (“capuchon” stage) [57]. Simultaneously, the number of GABAergic synapses from basket cells (BCs) begin to increase steeply, and the majority of perisomatic synapses switch from excitatory CF to inhibitory BC synapses from P9 to P15 (discussed later) [59, 60]. In the “dendritic” stage [57], CF synapses progressively translocate to growing PC dendrites [31, 32, 52, 61]. Relative extension of the CF in the molecular layer increases from 45 % at P12 to 70 % at P15 and 76 % at P20 [52, 59], and further increases thereafter [62]. In the dendritic stage, CF translocation thus proceeds rather slowly and follows dendritic extension with some time lag.

**Fig. 2:**
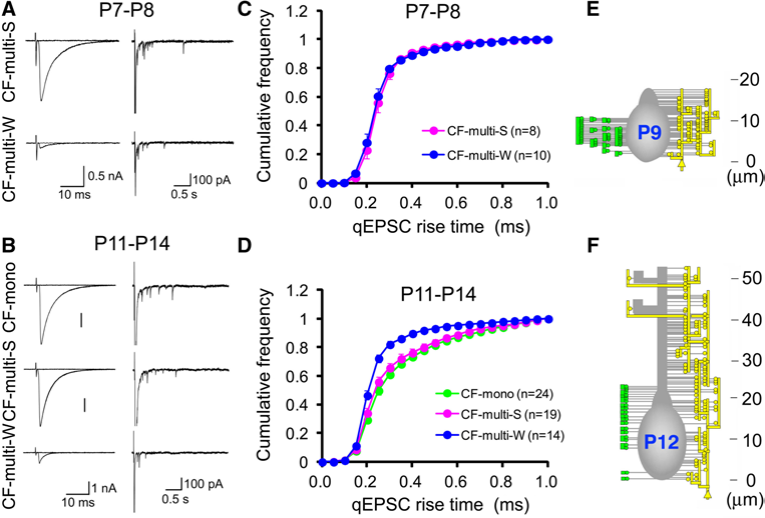
Translocation of the CF to PC dendrites. **a** (*left*) Representative traces of EPSCs evoked by stimulating CF-multi-S (*upper*) and CF-multi-W (*lower*) in the normal Ringer solution at P7–P8. (*right*) Asynchronous qEPSCs elicited in the Sr^2+^-containing solution. **b** Similar to (**a**), but those for stimulating CF-mono (*top*), CF-multi-S (*middle*) and CF-multi-W (*bottom*) at P11-P14. **c** Average cumulative histograms for the 10–90 % rise times of qEPSCs arising from CF-multi-S (*pink*, *n* = 8), and CF-multi-W (*blue*, *n* = 10) at P7–P8. **d** Average cumulative histograms for the 10–90 % rise times of qEPSCs arising from CF-mono (*green*, *n* = 24), CF-multi-S (*pink*, *n* = 19), and CF-multi-W (*blue*, *n* = 14) at P11–P14. **e** Innervation territories reconstructed from serial electron microscopy at P9. *Yellow lines* and *circles* on the *right side* of each PC represent the terminals formed by the predominant CF, while *green circles* on the *left side* represent terminals formed by weak CFs. **f** Similar to (**e**), but the representative reconstructed image at P12. Modified from Hashimoto et al. [52], with permission from Elsevier

When the CF translocation is in progress, most of the PCs are still innervated by multiple CFs. Hashimoto et al. [52] have revealed how the CF translocation is correlated with the functional differentiation and the elimination of redundant CFs by using electrophysiological and morphological techniques. The location of synapses along the somato-dendritic domains of PCs was estimated by analyzing the kinetics of EPSCs arising from single synaptic vesicles (i.e., quantal EPSCs) in CF terminals [52]. Because the kinetics of quantal EPSCs is distorted due to dendritic filtering, the incidence of quantal EPSCs with slower rise times is higher for translocating CFs than for CFs innervating perisomatic regions. Quantal EPSCs originating from particular CFs can be recorded in Sr^2+^ containing extracellular solution which causes asynchronous release of transmitter quanta following CF stimulation [49, 63] (Fig. 2a, b). CF-mediated EPSCs were recorded from PCs that were multiply innervated by single ‘‘strong’’ CFs (CF-multi-S) and one or a few ‘‘weak’’ CFs (CF-multi-W). At P7–P8, cumulative histograms for the 10–90 % rise time of quantal EPSCs arising from the strongest CF (CF-multi-S) and other weaker CFs (CF-multi-W) were identical (Fig. 2c). The incidence of quantal EPSCs with slower rise times began to increase for CF-multi-S from P9 to P10. The difference in the distribution of quantal EPSC rise times for CF-multi-S and for CF-multi-W became larger from P11 to P14 (Fig. 2d). The distribution of quantal EPSC rise times for CF-multi-S was identical to those for CFs of mono-innervated PCs (CF-mono). These results suggest that the strongest CF and other weaker CFs both innervate the PC soma at P7–P8, but only the most strengthened CFs begin to expand their innervation territories to dendrites from P9 to P10. Because the disparity among the synaptic strengths of multiple CF inputs already reaches a plateau at around P7–P8 [49], this result indicates that the functional differentiation of multiple CFs occurs on the PC soma during “pericellular nest” stage.

These electrophysiological results have been confirmed by detailed morphological analyses. A small amount of an anterograde tracer, BDA, was injected into the inferior olive to label a subset of CFs, and an antibody against VGluT2 was used to stain all CF terminals. When a PC had double-labeled CF terminals for BDA and VGluT2 and single-labeled ones for BDA, the PC was judged to be innervated by more than two CFs with distinct cellular origins in the inferior olive [52]. Serial electron microscopic analysis was performed in PCs innervated densely by BDA-labeled CFs that were presumed to be predominant “strong” CFs in individual PCs. At P9, some somatic spines formed asymmetrical synapses with CF terminals that were double-labeled for BDA and VGluT2 (yellow CF terminals in Fig. 2e), while other spines of the same PC formed asymmetrical synapses with CF terminals that were single-labeled for VGluT2 (green CF terminals in Fig. 2e). The double-labeled CF formed on average 57 % of terminals around the PC soma at P9 [52], which confirmed retrospectively that the double-labeled CF was indeed the strongest CF for the PC. This result indicates that all CF synapses, originating from the predominant CF and weaker CFs, are confined to the soma or the basal part of dendrites of PCs at P9 (Fig. 2e). At P12, proximal shaft dendrites were exclusively associated with the predominant CF, whereas the somata of PCs were contacted by both the predominant and other weaker CFs (Fig. 2f). Density of such synaptic terminals on the soma was greatly reduced by P15, indicating that massive elimination of somatic CF synapses occurs from P12 to P15. Synapse elimination during this postnatal period might result from a ‘non-specific’ removal of CF terminals around the PC soma without affecting CF terminals on PC dendrites. This may provide a basis for elimination of surplus CFs remaining on the PC soma and sparing the predominant CF innervating PC dendrites. While surplus CFs that have synapses only on the PC soma may be liable to ‘elimination signals’ and eliminated, the strengthened CF that forms synapses on PC dendrites may survive from such signals. This postnatal period largely overlaps with that for the late-phase elimination process (see Fig. 4). Taken together, these electrophysiological and morphological data indicate the following three points of CF synapse refinement. (1) Synaptic competition among multiple CFs occurs on the soma until around P7–P8, which almost corresponds to the “pericellular nest” stage. In this period, one CF forms aggregated terminals on the PC soma and becomes as the most predominant CF with strongest synaptic efficacy. (2) Then, in each PC, only the strongest CF (“winner” CF) translocates to PC dendrites after P9. And (3) the weaker CFs (“loser” CFs) remain innervating the soma of PCs (Fig. 1a, ~P12) but are finally eliminated. Very recently, Carrillo et al. [64] reported the results from two-photon multicolor vital imaging of CFs in developing mouse cerebellum in vivo. Their results largely confirm the conclusions derived from the electrophysiological and morphological data described above. Moreover, their data from in vivo time-lapse imaging have revealed that the motility of CF terminals on the soma is much higher than those on dendrites, and that the CF which has begun dendritic translocation indeed becomes the winner.

In parallel with the removal of CF synaptic terminals on the PC soma, GABAergic synapses are massively formed on PCs [59, 60]. PCs receive inhibitory synapses from BCs and stellate cells in the molecular layer. BC axons innervate the PC soma and form the pinceau organization at the axon initial segment of PCs, while stellate cells innervate dendrites of PCs [7]. In the developing cerebellum after removal of CF innervations from the PC soma, GABAergic BC synapses have been shown to be predominant synapses on the PC soma [59, 60]. Somatic innervation of BCs became obvious around P7 [59, 60], but until around P9 (the end of the pericellular nest stage), most perisomatic synapses were formed by CFs on somatic spines (CF-spine) [59]. The density of CF-spine synapses continuously decreased after P12–P20, whereas perisomatic synapses from BCs increased reciprocally. In transition, a substantial number of somatic spines which were originally innervated by CFs became innervated by BC synapses, or surrounded by Bergmann glial processes (free spine). By P20, BC-spine synapses and free spines disappeared, and BCs came to form synapses directly on the PC somata. Importantly, fragmental clusters of AMPA receptors were juxtaposed with those of GABA_A_ receptors at postsynaptic sites beneath single BG terminals on PC spines [59]. These lines of evidence suggest that some postsynaptic spines originally occupied by CFs are taken over by the BC synapses. The somatic free spines associated by glial processes might suggest the engulfment of CF terminals by the glia. The interactions among the CF, the BF, and the glia on PC spines are required for the switching of somatic synapses.

The factors controlling the CF translocation are not well known. In peroxisomal biogenesis factor 2 (Pex2) knockout mice, the CF innervation was largely confined around the PC soma even at P13 [65]. However, Pex2 mutant mice had several abnormalities in cerebellar development (e.g., GC proliferation, PC morphology, cerebellar foliation) other than CF translocation, and died before the third postnatal week. Thus, it is currently difficult to identify the roles of Pex2 in the CF translocation [65]. There are several mutant mice in which CF innervation extends to PC dendrites, but the height of CF innervation territory in the molecular layer is reduced when compared to control mice (diminished translocation) [e.g., neuron/glia specific focal adhesion kinase (FAK) knockout mice [66], myosin Va mutant mice [67], Cav2.1 mutant mice [68, 69], PC specific CIC-1 expressing mice [70] and Atxn1 mutant mice [71]]. Because the regression of CF territory is induced when neuronal activities are suppressed by tetrodotoxin [72] or NBQX [73] even in the adult cerebellum, diminished translocation observed in the several mutant mouse lines might be caused by altered activities of pre- and postsynaptic neurons. In addition to the diminished translocation, Cav2.1 mutant mice exhibited translocation of multiple CFs to PC dendrites [54]. This abnormality may be a consequence of the nonselective strengthening of multiple CFs in Cav2.1 mutant mice. Multiple CFs with synaptic strengths larger than a certain level might be permitted to extend their innervations to PC dendrites.

## Mechanisms underlying the early phase of CF elimination

In the mouse cerebellar slices, the average number of CFs that innervate individual PCs does not decrease but slightly increases from P3 to P6 when the functional differentiation of multiple CFs occurs [13]. The value begins to decrease from P7, just after the disparity among the strengths of multiple CF inputs reaches a plateau. Therefore, CF synapse elimination does not proceed in parallel with functional differentiation of multiple CFs, but starts after the strengthening of single CFs in individual PCs.

As we mentioned in the previous section, surplus CF elimination proceeds in at least two distinct phases: the early and the late phase of CF elimination. The early phase of CF elimination occurs from P7 to around P11 [13]. Mechanisms of the early-phase of CF elimination remain largely unknown. However, several lines of evidence suggest the importance of the neuronal activity for this event. Andjus et al. [74] demonstrated that the CF elimination was impaired by disrupting the normal firing pattern of CFs from P9 to P12 by administration of harmaline, which facilitated synchronous activities among inferior olivery neurons in rats. Assuming that the developmental course of CF synapse elimination is similar between rats and mice, this result suggests that the abnormal over-synchronized activation of CFs during the early phase impairs synapse elimination. Moreover, recent analyses indicate that the activity of the postsynaptic PC is crucial for the CF elimination. Lorenzetto et al. [70] generated transgenic mice expressing a chloride channel, ClC-1, specifically in PCs, and found the impairment of CF synapse elimination. In this transgenic mouse, the expression of CIC-1 became evident at P5–P9 and persisted into adulthood. Therefore, it is difficult to determine whether early- or late-phase of CF elimination is impaired in this transgenic mouse. Meanwhile, Hashimoto et al. [54] reported that synapse elimination starting at P7 was severely impaired in mutant mice with PC-selective deletion of P/Q type VDCC. These results collectively indicate that adequate activity of CFs and following Ca^2+^ influx into the postsynaptic PCs are critical for the early-phase CF elimination.

In addition, insulin-like growth factor I (IGF-1) is reported to be involved in the CF synapse elimination from P8 to P12 [75]. Local application of IGF-1 not only suppressed synapse elimination but also enhanced the amplitudes of the smaller CF-EPSCs of the multiply innervated PCs, suggesting that IGF-1 provides a trophic support to maintain CF synapses. It is possible that selective assignment of limited trophic factors, such as IGF-1, to the strengthened CF may occur in an activity-dependent manner, which may facilitate the survival of the winner CF and the elimination of the loser CFs.

## Mechanisms underlying the late phase of CF elimination

The late-phase of CF elimination starts at around P12 in mice [13]. While the molecular mechanisms for the early-phase elimination are largely unknown, those for the late phase elimination have been clarified to better extent. Recent analyses have demonstrated the crucial roles of the heterosynaptic regulations of the late-phase of CF elimination by PFs and GABAergic synapses. In the following section, we discuss the molecules whose manipulations have been reported to affect CF synapse elimination after P10. It should be noted that the beginning of the late phase of CF elimination varies by 1 or 2 days depending on experimental procedures (e.g., in vivo or in vitro, morphological or electrophysiological analysis), the area where PCs are sampled (e.g., vermis or hemisphere, ventral or dorsal side of the cerebellum [61]) and/or species (e.g., rat or mouse).

As mentioned in the “Introduction”, previous reports indicate that the late-phase of CF elimination is critically dependent on the proper formation of GCs and PFs. However, most of the animal models used in the previous experiments have severe abnormalities in cerebellar size, layer structure, PC spinogenesis and/or degeneration of GCs [40, 56, 76–79]. Therefore, it was not easy to determine which factors are essential for CF synapse elimination. The analysis of mutant mice deficient in the glutamate receptor δ2 subunit (GluD2) demonstrates that proper formation of PF to PC synapses is crucial for the late-phase of CF elimination that begins at around P12 [80, 81]. PFs form excitatory synapses on the spines of PCs’ distal dendrites. Each synapse is weak, but as many as 100,000–200,000 PF synapses are formed on a single PC. The deletion of GluD2 markedly impaired formation and stabilization of PF-PC synapses, but did not greatly affect gross anatomy of the cerebellum and morphology of PCs [82, 83]. Importantly, the GluD2 KO mice exhibited abnormalities in CF innervation patterns. As discussed in the previous section, weaker CFs innervate only around the PC soma in developing cerebellum of wild-type mice, and such a somatic CF innervation disappears at around P15. In adult GluD2 KO mice, multiple CF innervation was observed not only around the PC soma but also in PC distal dendrites where PFs normally form synapses [80, 81]. The CF innervation in distal dendrites are mainly formed by transverse collaterals [84] emerging from CFs that innervate neighboring and remote PCs. The aberrant dendritic innervation was also observed in the mice with hypogranular cerebellum generated by methylazoxy methanol acetate treatment [13] or mutant mice deficient in Cbln1 [85] that binds to GluD2 and stabilizes PF-PC synapses [86, 87]. Importantly, a similar aberrant dendritic innervation by CFs could be induced by deletion of GluD2 in adult mice, suggesting that the ectopic CF innervation of PC distal dendrites occurs not only during postnatal development but also in adulthood when PF-PC synapses are destabilized [88]. By contrast, the somatic multiple CF innervation observed in global GluD2 KO mice [80] was not induced by the deletion of GluD2 in adult mice [88]. This evidence suggests that the persistent somatic CF innervation is the consequence of impaired late-phase of CF elimination. These results indicate that PFs play two distinct roles in establishing CF innervation of PCs: (1) PFs restrict the sites of CF innervation to proximal dendrites of PCs; this function is seen not only during postnatal development but also in adulthood; and (2) PFs drive the late-phase of CF elimination by removing somatic CF synapses during postnatal development [88].

In mice deficient in the type 1 metabotropic glutamate receptor (mGluR1) or any of its downstream signaling molecules (Gαq, PLCβ4, PKCγ), the late-phase of CF elimination has been shown to be severely impaired [89–93]. The defect of CF synapse elimination in mGluR1 knockout mice was restored in mGluR1-rescue mice in which mGluR1a was introduced specifically into PCs of mGluR1 KO mice [94]. Regression of CF synapses was impaired in mice by PC-specific expression of a PKC inhibitor peptide [95]. Furthermore, impaired CF synapse elimination of PLCβ4 KO mice was confined to the rostral part of cerebellum where PCs predominantly express PLCβ4. [91, 96]. These lines of evidence indicate that the mGluR1 signaling cascade within PCs is essential for the late-phase CF synapse elimination.

Although mGluR1 is richly expressed at perisynaptic sites of PC dendritic spines facing PFs and CFs, mGluR1 can be activated readily by PF inputs [97–99], while hardly by CF inputs without blockade of glutamate transporters [100]. Furthermore, chronic blockade of NMDA receptors within the cerebellum have been shown to result in the impairment of CF synapse elimination [101] specifically in its later phase [102]. NMDA receptors are absent [89, 102, 103] or barely detectable [104] at PF and CF synapses of young PCs, but they are abundantly expressed at mossy fibers (MF) to GC synapses [102] in the developing cerebellar cortex. Thus, it is most likely that chronic NMDA receptor blockade in the cerebellum impairs the late phase CF elimination by suppressing the activation of NMDA receptors at MF-GC synapses [102]. These results strongly suggest that mGluR1 is activated at PF-PC synapses by neural activity along the MF-GC-PF pathway and plays a pivotal role in the late phase of CF elimination [102].

Recent analyses have revealed that GABAergic inhibition is also crucial for CF synapse elimination. Nakayama et al. [55] has recently reported that CF synapse elimination from P10 to P16, corresponding to a latter part of the early-phase and the entire late-phase elimination, is impaired in mice with a heterozygote deletion of the GABA synthesizing enzyme GAD67. This abnormality was also observed in the mutant mice in which GAD67 was selectively deleted from PCs and cerebellar interneurons, or in the wild-type mice that received a local and persistent application of a GAD blocker, 3-MP, into the cerebellum from P10. Moreover, the defect in CF synapse elimination in GAD67 heterozygote mice was restored by a local chronic application of a GABA_A_ receptor sensitizing agent, diazepam, to the cerebellar cortex. These results indicate that impaired CF synapse elimination in GAD67 heterozygote mice is the result of reduced activation of GABA_A_ receptors within the cerebellum. Miniature inhibitory postsynaptic currents (mIPSCs) with large amplitudes and fast rise times, which presumably derived from BC terminals on the PC soma, were greatly reduced in GAD67 heterozygote mice. When paired whole-cell recordings were made from a putative BC and a PC, the amplitude of unitary IPSCs of GAD67 heterozygote mice was reduced to about half of that of wild-type mice. Because of the reduced inhibition to the PC soma, stimulation of weak CF in GAD67 heterozygote mice induced significantly larger Ca^2+^ transients in the PC soma than in wild-type mice. While the size of somatic Ca^2+^ transients by activation of weak CF was much smaller than that by the strongest CF in wild-type PCs, weak CF could induce somatic Ca^2+^ transients that were comparable in amplitude to those elicited by the strongest CF activation in GAD67 heterozygote PCs. Consequently, the Ca^2+^ influx around somatic CF synapses might be large enough to overcome synapse elimination signals that otherwise prune most of the somatic CF synapses during the late phase CF elimination [55, 105].

A neurotrophin receptor, TrkB, is also suggested to be involved in CF synapse elimination that starts from around P10–P12 [106, 107]. It is currently unclear how TrkB is involved in CF synapse elimination. TrkB was reported to be expressed in the inferior olive during early postnatal days [108]. Sherrard et al. [109] demonstrated that the expression of truncated form of TrkB, a negative regulater of TrkB signaling, rose from P4 in the inferior olive and from P7 in the cerebellum. These data suggest that reduced activation of TrkB signaling might underlie the elimination of surplus CFs that starts several days later [108, 109]. Another interesting possibility is that TrkB may affect CF synapse elimination through promoting the maturation of inhibitory synaptic terminals. In TrkB mutant mice, the number of GABAergic terminals in cerebellar cortex was reduced [110], and the mIPSCs recorded from PCs had immature profiles in kinetics [106]. Previous reports have suggested that BDNF and TrkB have important roles in formation and maturation of GABAergic synapses in the cerebellum [111, 112]. It is therefore possible that the defect of the late-phase CF elimination in TrkB-deficient mice results indirectly from the impaired development of inhibitory neuronal circuits in the cerebellum.

**Fig. 3:**
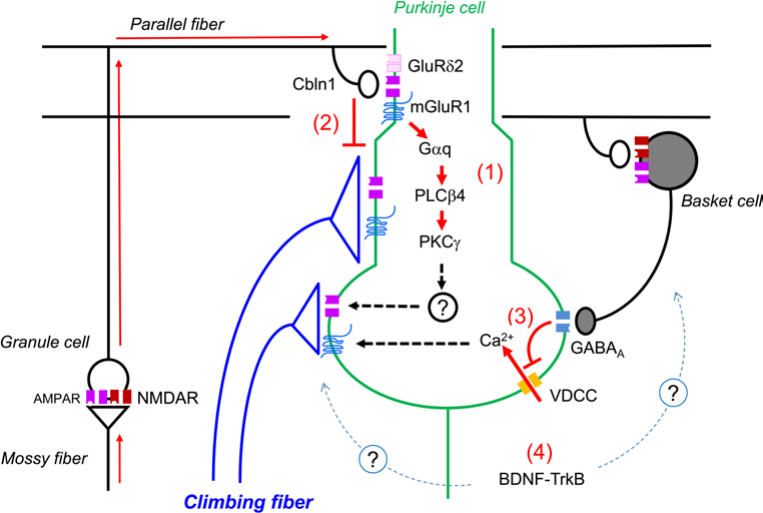
Mechanisms underlying the late-phase of CF elimination. The late-phase is dependent on heterosynaptic interactions between CF and PF synapses and between CF and BC synapses. (*1*) Neural activity along the MF-GC-PF pathway activates mGluR1 and downstream signaling cascades at PF-PC spines. (*2*) Stabilization of PF-PC synapses on spines of distal dendrites of PCs restrict the innervation sites of CFs to proximal dendrites. (*3*) BC terminals on the PC soma suppress the Ca^2+^ influx through activation of VDCCs. (*4*) BDNF-TrkB signaling might promote the maturation of GABAergic neurons and/or regression or maintenance of CF synapses. Modified from Kano et al. [93]

**Fig. 4:**
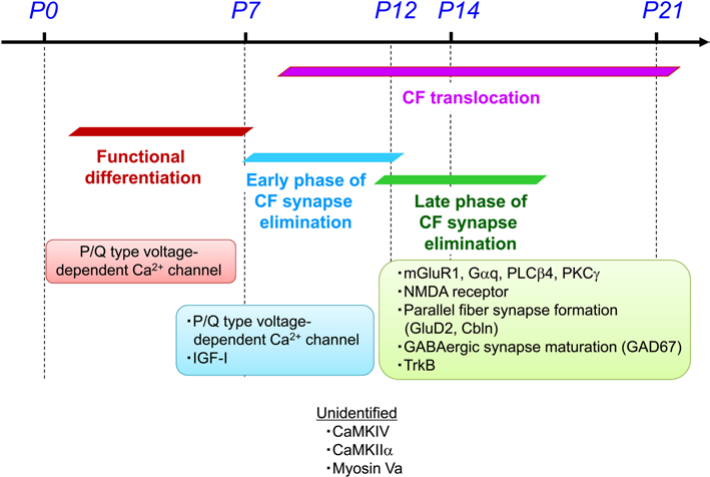
Molecular mechanisms for the postnatal refinement of CF to PC synapses. Modified from Kano and Hashimoto [14], with permission from Elsevier

The current hypothesis for the mechanisms underlying the late-phase CF elimination is illustrated in Fig. 3. First, activation of mGluR1 at PF-PC synapses is considered to drive the late-phase of CF synapse elimination (Fig. 3, 1). Activation of mGluR1 seems to occur at PF-PC synapses by neural activity along MF-GC-PF pathway that involves NMDA receptors at MF to GC synapses [102]. Second, stabilization of PF-PC synapses on spines of distal dendrites of PCs restricts the innervation territory of CFs to proximal dendrites of PCs (Fig. 3, 2). Reduced PF synapse formation by the lack of GluD2 or Cbln1 [80, 81, 85] or by suppression of GC genesis [13] inevitably causes ectopic CF synapse formation on distal dendrites of PCs and thereby results in the impairment of the late-phase of CF elimination. Third, maturation of GABAergic inhibitory synaptic transmission positively regulates the late-phase of CF elimination by facilitating the removal of CF synapses on the PC soma [55] (Fig. 3, 3). In addition, BDNF-TrkB signaling seems to influence CF synapse elimination either indirectly through promoting the maturation of GABAergic inhibitory circuitry or by directly regulating maintenance and elimination of CF synapses (Fig. 3, 4).

## Concluding remarks and Future perspectives

The scheme in Fig. 4 summarizes the processes for postnatal refinement of CF-PC synapses and their molecular mechanisms. The P/Q type VDCC in the postsynaptic PC is critical for the functional differentiation, the early phase of CF elimination and the CF translocation. Molecular mechanisms for these processes remain largely unknown, but signaling molecules activated by Ca^2+^ influx are potential candidates. While several reports suggest the importance of neuronal activity in these processes, activity patterns in immature cerebellar circuits have not been well studied. What patterns of neural activity are important? How are such activity patterns generated? How much activity is required for proper maturation of cerebellar circuits? These issues should be addressed in intact cerebella from living immature animals in vivo.

The late-phase of CF elimination is critically dependent on the mGluR1 signaling cascade in PCs and GABAergic inhibition to PC somata. It is currently unknown whether these two pathways work in parallel or if they ultimately converge on the common process for the late phase of CF elimination. In addition, the final mechanisms that execute morphological pruning of surplus CF synapses are also unknown. On this point, a previous report suggests that surplus CF synapses might be engulfed by other cells [113]. The CF branches that had a fragmented appearance on PC somata and shaft dendrites were associated with organelles that had lysosomal activity [113]. During postnatal development, retreating motor axons were engulfed by Schwann cells and removed from the neuromuscular junction [113, 114]. Contribution of glia is also suggested in the postnatal refinement of retio-geniculate synapses [115–117]. It is possible that waning CF synapses might be recognized by nearby glia for final digestion in a manner similar to the retreating motor axons at the neuromuscular junction.

As for other molecules potentially involved in CF elimination, null mutant mice lacking Ca^2+^/calmodulin-dependent kinase IV (CaMKIV) are reported to have persistent multiple innervations, but it is unclear what stage of the postnatal process is impaired [118]. It is also reported that null mutant mice deficient in α Ca^2+^/calmodulin-dependent protein kinase II (CaMKIIα) display multiple innervations at P21–P28, but this phenotype disappears in adulthood, suggesting delayed CF synapse elimination [119]. Such delayed synapse elimination is also reported in the hypothyroid rat [46] and the Dilute Neurological mouse [67]. The roles of these molecules in synapse elimination should be addressed in future studies.

The candidate genes are picked up by their spatial and temporal expression profiles during postnatal development, or differential expression between wild-type mice and mutant mice with impaired CF synapse elimination. The functions of these candidate genes in cerebellar development are normally checked by gene knockout or pharmacological manipulations. However, gene targeting generally requires significant efforts and time. Pharmacological manipulation of molecules in the brain in vivo accompanies the problem of drug specificity and the difficulty in persistent, local, and stable application of drugs to the developing brain whose size and structure change continuously. For high-throughput screening of candidate molecules, establishment of culture system for analyzing synapse elimination have been attempted [120–122]. In the organotipic olivocerebellar coculture system recently developed by Uesaka et al. [122], PCs are initially innervated by multiple CFs and progressively eliminated until DIV14–16. Importantly, the synapse elimination is dependent on GluD2, mGluR1 and NMDA receptors that are all essential for CF synapse elimination in vivo. The culture systems have strong advantages for exploring molecular mechanisms. Spatially and temporally controlled manipulations are possible in pharmacological experiments and in experiments with gene knockdown and overexpression. Moreover, it is also easy to manipulate multiple candidate factors. The development of culture systems will strongly facilitate the elucidation of molecular mechanisms for synapse elimination. Using this olivo-cerebellar coculture preparation, Mikuni et al. have reported very recently that the immediate early gene* Arc/Arg3.1* mediates the late phase of CF elimination [123].

## Abbreviations

BC: Basket cell
CF: Climbing fiber
EPSC: Excitatory postsynaptic current
GC: Granule cell
IPSC: Inhibitory postsynaptic current
MF: Mossy fiber
PF: Parallel fiber
PC: Purkinje cell
VDCC: Voltage-dependent Ca^2+^ channel
